# The Design and Impact of Teaching Kitchens and Hands-On Cooking Strategies on Diverse Populations: Increasing Evidence of Positive Effects and Proposed Future Directions

**DOI:** 10.3390/nu17233638

**Published:** 2025-11-21

**Authors:** David M. Eisenberg, Alexis Cole, Lorena S. Pacheco, Jennifer Massa, Aviad Haramati, Andrew A. Bremer

**Affiliations:** 1 Department of Nutrition, Harvard T.H. Chan School of Public Health, Boston, MA 02115, USA; lpacheco@hsph.harvard.edu (L.S.P.); jmassa@hsph.harvard.edu (J.M.); 2 Gerald J. and Dorothy R. Friedman School of Nutrition Science and Policy, Tufts University, Boston, MA 02111, USA; lexi.cole@tufts.edu; 3 Center for Innovation and Leadership in Education (CENTILE), Georgetown University School of Medicine, Washington, DC 20007, USA; haramati@georgetown.edu; 4 Office of Nutrition Research, National Institutes of Health, Bethesda, MD 20817, USA; andrew.bremer@nih.gov

## 1. Introduction

As rates of diet-related chronic diseases continue to rise—alongside the associated costs to healthcare systems, individual well-being, and environmental sustainability—there is an urgent need for innovative approaches to counter the harmful effects of our current pathogenic food environment. Teaching kitchens—which serve as learning laboratories for essential life skills including nutrition education, hands-on cooking instruction, enhanced physical activity, mindfulness training, and instruction in behavior change strategies—represent a promising solution for training a new generation of informed eaters, educators, and health professionals equipped to drive meaningful behavior change and foster health promotion, also known as salutogenesis.

In January 2022, the *Nutrients* Editorial Board issued a call for manuscripts for a new Special Issue focusing on the following question: “*How can health and wellness promotion strategies which include nutrition education alongside hands-on cooking be organized, evaluated, and optimized for maximal impact?*” [1]. This invitation resulted in the acceptance and publication of 36 articles which addressed the role of teaching kitchens and hands-on cooking in relation to a range of nutrition education-related interventions. The studies and review articles published in this Special Issue help answer questions related to the most effective ways to educate individuals, families, communities, and health professionals with respect to nutrition and food choices; provide evidence that these approaches predictably change behaviors and outcomes of interest; and highlight examples as to how these multi-disciplinary educational interventions have the potential to improve human and planetary health.

In this commentary, these articles have been grouped into three core categories—definitions and descriptions of teaching kitchens, evaluations of teaching kitchens as applied to distinct populations across multiple settings and selected “other” topics.

This invited commentary provides a summary overview of these 36 publications along with reflections on the following: (i) what has been observed and learned with respect to the development and application of these novel educational approaches across a range of populations and settings; (ii) what remains to be studied to refine and improve these approaches; and (iii) ways in which this emerging literature can inform global research and medical education relating to teaching kitchens, culinary medicine, lifestyle medicine, integrative medicine, the Food is Medicine (FIM) movement, whole-person health research, and the rapidly emerging United States (US)-based “Make America Healthy Again” (MAHA) movement. Importantly, the articles from this Special Issue, alongside the existing body of teaching kitchen literature described herein, confirm the emerging growth of research involving teaching kitchens and hands-on cooking as valuable adjuncts to nutrition education teaching methods and interventions.

This commentary highlights how health practitioners, educators, and policymakers are increasingly mobilizing to identify effective strategies—including the use of teaching kitchens—that enhance the health of individuals, communities, and the planet.

## 2. Descriptions of *Nutrients* Special Issue Articles

Between June 2023–June 2024, 36 articles were accepted and published in the *Nutrients* Special Issue. This Special Issue included 32 research articles, 2 review papers, and 2 “other” articles.

After reviewing all 36 articles, three core categories emerged: definitions and descriptions of teaching kitchens, evaluations of teaching kitchens as applied to distinct populations across multiple settings, and selected “other” topics. Articles about specific populations were subsequently divided into seven sub-categories—health professional trainees, adults with chronic disease, individuals facing social stressors, university students, employees, children, and others. Articles about selected other topics included the connection between teaching kitchens and agriculture and food systems, billing strategies, and strategies to enhance the solicitation of a patient’s food history.

**Figure 1:**
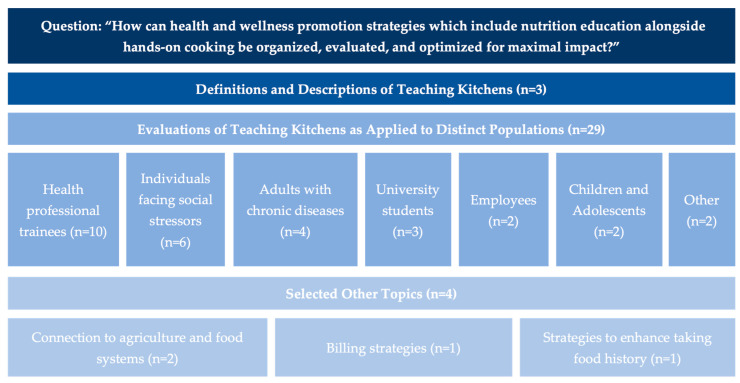
Articles in the *Nutrients* Special Issue arranged by core category (N = 36).

Figure 1 displays the core categories as they relate to the Special Issue prompt question: “How can health and wellness promotion strategies which include nutrition education alongside hands-on cooking be organized, evaluated, and optimized for maximal impact?”. Table 1 lists the articles by categories and sub-categories and includes a summary of the main findings of each article. Further details about each of these articles, including the population studied, sample size, study design, outcome(s) of interest, intervention, and key findings, are described in Supplementary Table S1.

All 36 papers add to the literature base demonstrating evidence for and strategies to organize, evaluate, and optimize health and wellness programs for maximal impact, especially through the combination of nutrition education with hands-on cooking. Several of these papers stand out as particularly innovative and have the potential to shape future efforts related to teaching kitchens, culinary medicine, whole-person health, FIM, and other lifestyle-based interventions. The authors describe a subset of these articles in further detail below.

## 3. Selected Articles

### 3.1. Perspective: Teaching Kitchens: Conceptual Origins, Applications and Potential for Impact Within Food Is Medicine Research [2]

This paper frames the entirety of the Special Issue, as it grounds the topic of teaching kitchens in terms of their origins, evidence, and structure, as well as opportunities for future research and practice. The paper makes the case that hands-on strategies (i.e., teaching kitchens) that train individuals in nutrition, mindfulness, movement, and behavior change have the potential to improve health outcomes, address social determinants of health, reduce barriers to healthy eating patterns, and slow rising healthcare costs. Teachings from Traditional Chinese Medicine—which tout the superiority of prevention over intervention and the influence of dietary, movement, and thought patterns on overall well-being—can be applied to modern medicine through teaching kitchens. Because teaching kitchens share many synergies and applications to fields such as culinary medicine, integrative medicine, lifestyle medicine, and FIM—and with the growing national attention in these complementary fields—there are countless opportunities for continued research and investment to demonstrate the potential economic, health, and social benefits of teaching kitchens as learning laboratories of the future.

### 3.2. Characteristics of Current Teaching Kitchens: Findings from Recent Surveys of the Teaching Kitchen Collaborative [3]

This paper describes common characteristics of teaching kitchens from Teaching Kitchen Collaborative members. Teaching kitchen programs are commonly delivered through the healthcare system, universities, and community-based organizations through a variety of modalities, including mobile carts, pop-up kitchens, pod kitchens, built-in-kitchens, and/or virtual settings. Teaching kitchens serve a range of individuals, including patients, practicing health professionals and trainees, employees, children, older adults, low-income individuals, and others, and are typically directed and/or facilitated by physicians, registered dietitians, chefs, and/or administrative directors or managers. Teaching kitchens offer multiple operational functions, with the majority of programs delivering culinary skills training and nutrition education and providing mindfulness training. Multiple funding streams exist to support teaching kitchens, including philanthropy, participant dues, organizational support, insurance, corporate sponsorship, and others, with most programs relying on more than one source. Future research should focus on best practices in program design, opportunities to integrate with the growing FIM movement, sustainable funding mechanisms, and—importantly—ways to better serve teaching kitchen participants.

### 3.3. “Zoom”ing to the Kitchen: A Novel Approach to Virtual Nutrition Education for Medical Trainees [12]

This paper addresses an important gap in nutrition education delivery by evaluating the effectiveness of a virtually delivered, single-session nutrition class—which included a hands-on teaching kitchen component—on students’ nutrition-related knowledge, attitudes, and behaviors. This course was delivered to physician assistant students and suggests that as little as one course may lead to persistent nutrition knowledge increases and confidence in nutrition counseling. This study indicates that even small doses of nutrition education, delivered virtually, can be effective in improving health professional trainees’ nutrition-related knowledge and skills, paving a path to scalability and sustainability.

### 3.4. Impact of Culinary Medicine Course on Confidence and Competence in Diet and Lifestyle Counseling, Interprofessional Communication, and Health Behaviors and Advocacy [13]

This study addresses three prominent topics in the teaching kitchen literature: the effectiveness of a culinary medicine elective on improving health professional trainees’ confidence and competence in food and nutrition counseling and knowledge, the impact of program delivery setting—in-person versus virtual—on participant outcomes, and differences in counseling confidence for students that enrolled in the elective culinary medicine course versus those that did not. An eight-week (24 h) culinary medicine course significantly improved health professional trainees’ confidence and competence in food and nutrition counseling, interprofessional communication skills, and healthy meal preparation. These findings hold constant whether the course was delivered in-person or online. Finally, this study demonstrated that medical students that participated in this culinary medicine course expressed significantly higher confidence in delivering nutrition and physical activity counseling to patients compared to students that did not take the course.

### 3.5. Assessing Acceptability: The Role of Understanding Participant, Neighborhood, and Community Contextual Factors in Designing a Community-Tailored Cooking Intervention [20]

This paper points to the importance of incorporating community members’ perspectives when designing effective teaching kitchen programs in order to best meet the needs of the target population. Participants in this study indicated that effective cooking interventions must address the following: (i) barriers to cooking (e.g., transportation, family and caregiving, time, household eating patterns, etc.); (ii) motivators to cooking (e.g., family, health, enjoyment, caregiving, etc.); (iii) applicable strategies (e.g., social support, meal planning, food shopping behaviors, etc.); (iv) neighborhood-based factors (e.g., safety, grocery store access, gentrification, stigma, etc.); and (v) intervention acceptability (e.g., influences to participate, delivery methods, recruitment strategies, etc.). By integrating community-informed insights during the planning phase, teaching kitchen and hands-on cooking programs are more likely to yield meaningful and sustainable outcomes for both researchers and participants.

### 3.6. Promoting Nutrition and Food Sustainability Knowledge in Apprentice Chefs: An Intervention Study at the School of Italian Culinary Arts—ALMA [26]

Chefs play an important role in individual and societal consumption patterns. While many culinary students have a moderate understanding of nutrition concepts, knowledge about sustainability concepts is lacking. This paper presents findings that an educational intervention which incorporates both nutrition and sustainability topics leads to significant improvements in students’ planetary health knowledge and small, but meaningful improvements in nutrition knowledge, suggesting that a sustainability-focused curriculum could drive improvements in global menus and dining patterns. Enhanced versions of the questionnaire developed for this study may also drive further assessments of culinary trainees’ health and sustainability knowledge and skills in both research and practice.

### 3.7. University Students as Change Agents for Health and Sustainability: A Pilot Study on the Effects of a Teaching Kitchen-Based Planetary Health Diet Curriculum [27]

In the face of both the global diet-related disease epidemic and climate crisis, this paper introduces an innovative approach to preparing future leaders in food and health. Through a university elective course delivered in a teaching kitchen setting, students from multiple disciplines engaged with planetary health diet concepts integrated into each lecture. Following the course, students showed significant improvements in planetary health diet literacy, suggesting that a hands-on, sustainability-focused nutrition curriculum can effectively influence knowledge, attitudes, and behaviors around healthy, sustainable cooking and eating practices.

### 3.8. Effect of the Emory Healthy Kitchen Collaborative on Employee Health Habits and Body Weight: A 12-Month Workplace Wellness Trial [29]

Worksite wellness programs have the potential to improve employees’ quality of life and overall health. This paper describes a 12-month teaching kitchen program which incorporated didactic, hands-on, and health coaching strategies to support a cohort of motivated individuals at an academic medical center. A multidisciplinary team led training sessions in culinary arts, mindful eating, yoga, exercise, stress resilience, and ethnobotany. Following the intervention, participants demonstrated improvements in diet quality, confidence in trying novel foods, mindful eating habits, and physical activity. This study demonstrates that engaging a multidisciplinary team as part of a workplace wellness strategy may lead to employees’ self-reported health behaviors.

### 3.9. The Role of Agricultural Systems in Teaching Kitchens: An Integrative Review and Thoughts for the Future [35]

This paper explores the intersections between agriculture, healthcare, culinary arts, nutrition science, and public health and highlights case studies where these often-siloed disciplines have come together to advance both human and planetary health. Increasingly, chefs, physicians, dietitians, and farmers are finding opportunities to collaborate to educate patients, communities, students, and medical trainees about the interconnectedness of food production, distribution, preparation, and consumption and the physiological and ecological consequences of each of these processes. Showcasing these linkages through teaching kitchen programs can elevate the roles of farmers and chefs within the growing Food is Medicine and culinary medicine movements, reinforcing their contributions to a more integrated and sustainable approach to health.

### 3.10. Culinary Medicine eConsults Pair Nutrition and Medicine: A Feasibility Pilot [36]

Enhancing referral pathways within healthcare teams can significantly improve patient access to essential services, including nutrition counseling and support. This paper presents an innovative pilot clinical workflow in which primary care physicians referred patients for a culinary medicine eConsult, delivered collaboratively by a physician-dietitian team. Most of these eConsults were successfully billed as reimbursable services, demonstrating the potential for a viable, scalable, and sustainable payment model—one that both compensates healthcare providers and expands patient access to nutrition counseling. Following the publication within the *Nutrients* Special Issue, the complete study paper illustrates the impact of this innovative culinary medicine service line which offers personalized food-based support while leveraging community kitchens as clinical sites through strategic partnerships [38]. The program’s early successes highlight its potential for broader adoption and phased growth of food as medicine initiatives.

### 3.11. Standard Patient History Can Be Augmented Using Ethnographic Foodlife Questions [37]

Typical food intake questionnaires are often perceived as clinical, impersonal, and disconnected from the lived experiences of patients. Yet, food is deeply tied to culture, emotion, and identity—far more than just its nutritional components. To enhance patient engagement, this paper proposes an expanded food history framework grounded in ethnographic methods, designed to explore individuals’ relationships with food and their underlying motivations. By enriching food history intake in this way, healthcare providers can foster deeper trust and open dialogue with patients, potentially leading to more meaningful and lasting improvements in health behaviors.

## 4. Discussion

These 36 articles add to the growing body of evidence demonstrating the value and applicability of teaching kitchens and other hands-on cooking experiential interventions to a wide range of populations across many settings. Outside of the publications from this *Nutrients* Special Issue, several other notable publications exist describing the role of teaching kitchens and hands-on cooking strategies in other settings and for other populations. These publications are described in Supplementary Table S2. While these and the articles in Table 1 offer a significant description of the state of the evidence on teaching kitchens, this paper is not a meta-analysis of the entirety of the teaching kitchen literature base. Rather, this paper represents a synthesis of the contents of the *Nutrients* Special Issue, plus the co-authors’ shared expertise and opinion regarding other publications of interest.

Together, these articles suggest that teaching kitchens and other multi-disciplinary educational interventions which include hands-on cooking may positively impact the populations displayed in Table 2.

It is the applicability of teaching kitchen-related interventions to so many populations that suggests that these emerging educational interventions warrant further refinement, replication, and formal evaluation.

The *Nutrients* Special Issue, alongside the additional articles cited above, provides an overview of more than ten years of collective work related to the scientific evaluation of teaching kitchens and culinary medicine and their potential for impact on individual and public health. Given the available evidence from this body of literature, several questions arise which can guide future research.

## 5. Future Research and Educational Opportunities

Further studies should investigate gaps in the evidence, such as the correct ‘dose’ and duration of a teaching kitchen intervention; minimal educational components of a teaching kitchen curriculum which will result in predictable behavior change; predictors of health-enhancing behavior change based on data from additional prospective teaching kitchen-related educational interventions; patient populations that should be prioritized; the integration of teaching kitchen programs with GLP-1 therapies to support the transition on and off of these intensive and expensive medications; cost-effectiveness analyses for specific patient populations and payer coverage by line of business; strategies whereby teaching kitchen instructors from a range of disciplines (e.g., dietitians, physicians, nurses, chefs, exercise and mindfulness instructors, behavior change experts, etc.) are trained, evaluated and “certified”; and the minimum necessary level of training required for medical students, physician trainees, and practicing clinicians to recommend and refer patients to teaching kitchen or other lifestyle medicine-based therapies.

In addition to these research gaps, further attention should be directed to several emerging themes and logistical considerations related to teaching kitchens and culinary medicine programs. First, comparative analyses of in-person versus virtual teaching kitchen models are needed to determine which components are best delivered in each format and how accessibility and engagement differ across populations and platforms. Second, there is an urgent need for cost modeling studies to determine the minimum financial investment required to achieve meaningful outcomes—whether in clinical, educational, or community-based settings. Third, future studies of FIM interventions should collect data on cooking confidence, skills, and frequency, as these may positively correlate (or potentially act as a confounding variable) with greater positive health outcomes (i.e., patients that receive culinary coaching or support in addition to produce prescriptions or medically tailored groceries may demonstrate greater health benefits compared to those who do not). Fourth, the development of robust and validated theoretical frameworks to explain the mechanisms through which teaching kitchen curricula impact behavior, clinical outcomes, and systems-level change remains a significant unmet need. Fifth, future research should explore predictors of successful intervention response, including sociodemographic factors, health status, baseline self-efficacy, and readiness for change. Lastly, well-designed longitudinal studies are required to determine the optimal follow-up duration for capturing both short- and long-term impacts of multidisciplinary teaching kitchen interventions. These should be conducted across diverse settings and populations to build a more generalizable evidence base and to guide the strategic implementation of these programs at scale.

Additionally, as evidence increases that teaching kitchens have the potential to impact behavior change, clinical outcomes, and the cost of medical care, it will become essential to establish criteria whereby teaching kitchen facilities, instructors, and relevant teaching kitchen educational ensembles can be credentialed, as third-party payers will likely require such standardization and accreditation.

The imperative to incorporate teaching kitchens into the medical curriculum can also be viewed as a catalyst for improving learning and work environments and can contribute to enhanced well-being of health professionals, faculty, and trainees. As the concept of *Human Flourishing* has been expanded to medical education to create a more conducive environment for learning and operationalized further to provide trainees with key skills, teaching kitchens can play a vital role by providing a unique opportunity to ‘Nourish to Flourish’ [39,40,41]. The comprehensive approach to flourishing in medical school—and in life—involves aligning key domains of one’s life, such as meaning, purpose, characters, values, and relationships; increasing physical activity; adopting mindfulness practices; and choosing better nourishment through an understanding of food and food choices; all of which can be fostered together through the use of teaching kitchens as classrooms.

This line of ongoing inquiry is additionally supported by the National Institutes of Health’s (NIH’s) Office of Nutrition Research (ONR). ONR is currently actively involved with myriad culinary medicine and teaching kitchen activities, both with the extramural research community as well as on the NIH campus itself. The ONR Teaching Kitchen Program—which currently serves The Children’s Inn at NIH which is planned to expand to serve the NIH Clinical Center and campus as a whole is highlighted in the Office’s current strategic plan [42,43]. Culinary medicine and teaching kitchen activities are also rooted within an approved NIH concept for comprehensive FIM Networks or Centers of Excellence [44]. These topics also align well with the recent HHS Press Release and Editorial calling for enhanced nutrition education requirements for physicians [45,46,47].

## 6. Conclusions

This is the first paper to our knowledge that includes many—if not most—of the relevant published articles relating to teaching kitchens, as well as other strategies that include hands-on cooking, and their potential to enhance nutrition education, clinical care, and management of selected patient populations. There is increasing evidence that these approaches can inform and, potentially, be incorporated into future educational, research, clinical, and policy recommendations.

We must consider and think of teaching kitchens in the context of current US and global trends relating to culinary medicine, lifestyle medicine, whole-person health, FIM, and the US-based MAHA movement. As healthcare leaders and practitioners, health plans, community organizers, policymakers, and researchers continue to invest in and investigate the effectiveness of these programs, it will be critical to integrate experts in teaching kitchen design, implementation, and evaluation to optimize health-promoting strategies for individuals, communities, and health systems.

## Figures and Tables

**Table 1:** *Nutrients* Special Issue articles arranged by core category *.

Articles by Core Category	Brief Summary
Definitions	
Perspective: Teaching Kitchens: Conceptual Origins, Applications and Potential for Impact within Food Is Medicine Research [2] Eisenberg DM, Pacheco LS, McClure AC, McWhorter JW, Janisch K, Massa J	This paper describes the **fundamental principles of teaching kitchens**, their conceptual origins, and the growing body of evidence demonstrating their effectiveness for various patient populations. Further, it summarizes their anticipated growth trajectory, relevance to other food- and nutrition-related fields of study, variations in teaching kitchen design, and thoughts for future research and practice.
Characteristics of Current Teaching Kitchens: Findings from Recent Surveys of the Teaching Kitchen Collaborative [3] Badaracco C, Thomas OW, Massa J, Bartlett R, Eisenberg DM	This paper describes the **settings, participants, models, functions, personnel, and funding sources** of dozens of existing teaching kitchens and makes recommendations for the continued improvement, expansion, and long-term sustainability of teaching kitchen programs.
Culinary Medicine or Culinary Nutrition? Defining Terms for Use in Education and Practice [4] Croxford S, Stirling E, MacLaren J, McWhorter JW, Frederick L, Thomas OW	This paper proposes an introductory model demonstrating the interrelationship between **culinary nutrition terms** and indicates that multidisciplinary collaboration amongst individuals trained in culinary arts, nutrition, health, and education underpins deployment of effective culinary nutrition interventions.
**Populations**	
*Health professional trainees*	
Development of a Culinary Medicine Curriculum to Support Nutrition Knowledge for Gastroenterology Fellows and Faculty [5] Lindsay KL, Kennedy J, Kim D, Kalra A, Parekh NK	This study presents positive findings on the acceptability and relevance of a tailored four-module culinary medicine curriculum delivered through a virtual platform by a chef and registered dietitian for **gastroenterology fellows and faculty**.
Addressing the Gap of Nutrition in Medical Education: Experiences and Expectations of Medical Students and Residents in France and the United States [6] Thircuir S, Chen NN, Madsen KA	This study illustrates the importance of integrating interdisciplinary and systems-based perspectives in nutrition and hands-on culinary teaching to bridge the gap between current **medical school training** and students’ perceived need to receive enhanced nutrition education in medical training.
Eat to Treat: The Methods and Assessments of a Culinary Medicine Seminar for Future Physicians and Practicing Clinicians [7] Donovan K, Thomas OW, Sweeney T, et al.	This study highlights the effectiveness of a six-module hands-on culinary medicine program facilitated by a registered dietitian to improve **medical students’ and practicing clinicians’** nutrition knowledge, self-efficacy, and practice-based confidence.
Comparison of Effectiveness regarding a Culinary Medicine Elective for Medical Students in Germany Delivered Virtually versus In-Person [8] Böttcher S, Schonebeck LJ, Drösch L, et al.	This study describes a seven-module culinary medicine elective and demonstrates that virtual and in-person teaching kitchen programs are equally effective modalities by which to deliver nutrition education and culinary training to **medical students**.
Cooking up Change: DEIB Principles as Key Ingredients in Nutrition and Culinary Medicine Education [9] Ring M, Ai D, Maker-Clark G, Sarazen R	This paper investigates the current landscape of the topics of diversity, equity, inclusion, and belonging in existing **food, nutrition, and health training programs**, and proposes a three-tier “Checklist for Culturally Competent Education in Nutrition.”
Impact of a Teaching Kitchen Curriculum for Health Professional Trainees in Nutrition Knowledge, Confidence, and Skills to Advance Obesity Prevention and Management in Clinical Practice [10] Thang CK, Guerrero AD, Garell CL, et al.	This paper demonstrates that the Upstream Obesity Solutions curriculum delivered to a variety of health professional trainees—**medical students, dental students, nursing students, and pediatric residents**—over varying course durations is effective in improving confidence in counseling, nutrition knowledge and literacy, and cooking skills.
Empowering Future Physicians and Communities on Chicago’s South Side through a 3-Arm Culinary Medicine Program [11] Maker-Clark G, McHugh A, Shireman H, et al.	This study demonstrates that an eight-session culinary medicine elective improves **fourth-year medical students’** confidence in nutrition counseling and personal dietary habits, improves community members’ confidence in chronic disease management and cooking skills, and is well-perceived by middle school participants.
“Zoom”ing to the Kitchen: A Novel Approach to Virtual Nutrition Education for Medical Trainees [12] Charles JA, Wood NI, Neary S, et al.	This study presents findings from a three-hour interactive virtual culinary medicine curriculum for **first-year physician assistant students** and demonstrates effectiveness in improving knowledge, attitudes, and confidence in nutrition counseling.
Impact of Culinary Medicine Course on Confidence and Competence in Diet and Lifestyle Counseling, Interprofessional Communication, and Health Behaviors and Advocacy [13] Brennan BR, Beals KA, Burns RD, et al.	This study highlights the effectiveness of an eight-week culinary medicine elective on improving **medical and health professional students’** confidence in diet and lifestyle counseling, interprofessional communication, and healthy meal preparation, and demonstrates that these findings are uniform across both in-person and virtual teaching environments.
Experiential Culinary, Nutrition and Food Systems Education Improves Knowledge and Confidence in Future Health Professionals [14] Shafto K, Vandenburgh N, Wang Q, Breen J	This study demonstrates that a six-week experiential food systems and culinary nutrition course for **health sciences graduate students** improves knowledge and attitudes about food skills, food systems, and chronic disease conditions as they relate to basic nutrition concepts.
*Adults with chronic diseases*	
An Intensive Culinary Intervention Programme to Promote Healthy Ageing: The SUKALMENA-InAge Feasibility Pilot Study [15] Domper J, Gayoso L, Goni L, et al.	This study suggests that a four-week culinary intervention for **older adults with overweight or obesity** leads to improvements in Mediterranean diet adherence, cooking confidence, weight, BMI, and waist and hip circumference compared to a nutrition education intervention alone.
Survivors Overcoming and Achieving Resiliency (SOAR): Mindful Eating Practice for Breast Cancer Survivors in a Virtual Teaching Kitchen [16] Huang S, Riccardi D, Pflanzer S, et al.	This study demonstrates that a nine-week virtual teaching kitchen intervention delivered by a multidisciplinary team leads to improvements in mindful eating behaviors for **survivors of breast cancer**.
Enhancing Chronic-Disease Education through Integrated Medical and Social Care: Exploring the Beneficial Role of a Community Teaching Kitchen in Oregon [17] Tanumihardjo JP, Davis H, Zhu M, On H, Guillory KK, Christensen J	This study highlights that a community teaching kitchen designed for actively engaged **adult patients with one or more chronic diseases** may lead to significant clinical improvements such as blood sugar and blood pressure control and help address unmet social needs, including reducing barriers to food access.
Redesigning Recruitment and Engagement Strategies for Virtual Culinary Medicine and Medical Nutrition Interventions in a Randomized Trial of Patients with Uncontrolled Type 2 Diabetes [18] McGuire MF, Chen PM, Smith-Morris C, et al.	This study describes barriers and facilitators to patient engagement in virtually delivered culinary medicine and medical nutrition therapy interventions for patients with **uncontrolled type 2 diabetes and food insecurity**.
*Individuals facing social stressors*	
Evaluation of the Effectiveness of a Bilingual Nutrition Education Program in Partnership with a Mobile Health Unit [19] French ML, Christensen JT, Estabrooks PA, et al.	This study reports on a bilingual nutrition education program with a mobile health unit designed for **low-income and underserved community residents** and indicates that this program achieved effectiveness in improving health and behavioral outcomes, especially for those most at risk.
Assessing Acceptability: The Role of Understanding Participant, Neighborhood, and Community Contextual Factors in Designing a Community-Tailored Cooking Intervention [20] Farmer N, Tuason R, Middleton KR, et al.	This study demonstrates that engaging participant perspectives—**African American adults with cardiovascular disease risk factors and low food access**—increases understanding of the barriers, motivators, environmental factors, and acceptability of health-focused interventions in community-based participatory research.
Food Is Medicine for Individuals Affected by Homelessness: Findings from a Participatory Soup Kitchen Menu Redesign [21] Wetherill MS, Caywood LT, Hollman N, et al.	This study describes a participatory-based approach to redesign a food shelter menu for **individuals experiencing homelessness** by assessing participant nutritional needs and taste preferences and responding with strategic menu guidelines.
Examination of an Online Cooking Education Program to Improve Shopping Skills, Attitudes toward Cooking, and Cooking Confidence among WIC Participants [22] Herman DR, Kimmel R, Shodahl S, Vargas JH	This study highlights that a cooking education curriculum delivered virtually to **WIC participants** leads to improvements in attitudes towards and confidence in cooking skills and cooking healthfully on a budget.
Cross-Sector Partnerships for Improved Cooking Skills, Dietary Behaviors, and Belonging: Findings from a Produce Prescription and Cooking Education Pilot Program at a Federally Qualified Health Center [23] Ylitalo KR, Janda KM, Clavon R, et al.	This study demonstrates that a six-week ‘food as medicine’ produce prescription program combined with hands-on cooking education for **patients of a federally qualified health center** leads to improvements in cooking self-efficacy, diet-related disease management, and daily vegetable intake.
Nourishing Conversations: Using Motivational Interviewing in a Community Teaching Kitchen to Promote Healthy Eating via a Food as Medicine Intervention [24] Temelkova S, Lofton S, Lo E, Wise J, McDonald EK 4th	This paper describes the benefits of motivational interviewing techniques in both individual and group settings to drive behavior change for **low-income individuals** in the context of a six-week cooking and nutrition course.
*University students*	
Impact of a Food Skills Course with a Teaching Kitchen on Dietary and Cooking Self-Efficacy and Behaviors among College Students [25] French CD, Gomez-Lara A, Hee A, et al.	This study demonstrates that a 14-week food skills course which includes both didactic and hands-on teaching delivered to **college students** leads to improvements in healthy cooking and eating behaviors and self-efficacy compared to a control group.
Promoting Nutrition and Food Sustainability Knowledge in Apprentice Chefs: An Intervention Study at The School of Italian Culinary Arts—ALMA [26] Franchini C, Biasini B, Giopp F, Rosi A, Scazzina F	This study describes an education intervention on nutrition and food sustainability knowledge for **culinary students** and demonstrates that the curriculum is effective in improving students’ knowledge and translation to menu design.
University Students as Change Agents for Health and Sustainability: A Pilot Study on the Effects of a Teaching Kitchen-Based Planetary Health Diet Curriculum [27] Rosenau N, Neumann U, Hamblett S, Ellrott T	This study indicates that a seven-week planetary health diet elective delivered through a teaching kitchen setting to **university students** is feasible, well-regarded by participants, and leads to improvements in planetary health diet literacy.
*Employees*	
Cultivating Healthier Habits: The Impact of Workplace Teaching Kitchens on Employee Food Literacy [28] Daker R, Challamel G, Hanson C, Upritchard J	This study reports on the impact of an in-person and virtual workplace teaching kitchen on improving food literacy for **employees at a multinational technology company** and describes engagement strategies for increasing teaching kitchen participation.
Effect of the Emory Healthy Kitchen Collaborative on Employee Health Habits and Body Weight: A 12-Month Workplace Wellness Trial [29] Bergquist SH, Wang D, Fall R, et al.	This study presents findings that a 12-month employer-based teaching kitchen program delivered by a multidisciplinary team leads to significant improvements in healthy eating behaviors and cooking confidence for **employees at risk of or experiencing chronic diseases**.
*Children and Adolescents*	
Cooking Skills, Eating Habits and Nutrition Knowledge among Italian Adolescents during COVID-19 Pandemic: Sub-Analysis from the Online Survey COALESCENT (Change amOng ItAlian adoLESCENTs) [30] Marconi S, Covolo L, Marullo M, et al.	This study demonstrates the positive correlation between cooking skills, nutrition knowledge, and healthy eating behaviors—especially reduced consumption of ultra-processed foods—in **Italian adolescents**.
A Specific Carbohydrate Diet Virtual Teaching Kitchen Curriculum Promotes Knowledge and Confidence in Caregivers of Pediatric Patients with Inflammatory Bowel Disease [31] Rivera N, Nguyen K, Kalami V, et al.	This study describes a 90 min virtual teaching kitchen session for **caregivers of pediatric patients with inflammatory bowel disease** and suggests that hands-on teaching may improve knowledge and attitudes toward a specific carbohydrate diet.
*Others*	
Feasibility of a Community Healthy Eating and Cooking Intervention Featuring Traditional African Caribbean Foods from Participant and Staff Perspectives [32] Moore SG, Kundra A, Ho P, Bissell E, Apekey T	This study explores the benefits of integrating participant perspectives—**individuals with African Caribbean backgrounds**—during program design to develop culturally adapted nutrition interventions which may help increase familiarity and acceptability of the foods offered.
Expectations from a Home Cooking Program: Qualitative Analyses of Perceptions from Participants in “Action” and “Contemplation” Stages of Change, before Entering a Bi-Center Randomized Controlled Trial [33] Polak R, Finkelstein A, Budd MA, et al.	This study delineates between “action” and “contemplation” stages of change related to home cooking behaviors for **individuals with overweight** and describes differences in concerns and expectations about health and lifestyle goals.
**Others**	
*Connection to agriculture and food systems*	
Teaching Kitchens and Culinary Gardens as Integral Components of Healthcare Facilities Providing Whole Person Care: A Commentary [34] Fals AM, Brennan AM	This paper explores the potential value of **embedding culinary gardens within clinical practice**, especially in the context of pediatric obesity management, and presents directions for incorporating teaching kitchens and culinary gardens within both in-person and virtual shared medical appointments to advance value-based care.
The Role of Agricultural Systems in Teaching Kitchens: An Integrative Review and Thoughts for the Future [35] Cole A, Pethan J, Evans J	This paper highlights **synergies between health practitioners, chefs, and farmers** and presents case studies wherein multidisciplinary teams collaborate to advance individual and community health, food is medicine initiatives, and broader planetary health objectives.
*Billing strategies*	
Culinary Medicine eConsults Pair Nutrition and Medicine: A Feasibility Pilot [36] Albin JL, Siler M, Kitzman H	This study describes how innovations in clinical workflows can facilitate referrals to a multidisciplinary culinary medicine team, **enhancing the delivery of personalized nutrition guidance to patients** and strategies for which these services can be routinely billed.
*Collecting food history*	
Standard Patient History Can Be Augmented Using Ethnographic Foodlife Questions [37] Lee JJ, McWhorter JW, Bryant G, Zisser H, Eisenberg DM	This paper proposes a **novel methodology by which to collect a patient’s food history** to increase patient engagement, improve provider understanding, and enhance patient–provider trust.

* Additional details about each article can be found in Supplementary Table S1.

**Table 2:** Populations for which teaching kitchens may offer benefits, based on the existing literature.

Health Professionals and Trainees	Adults with Chronic Diseases	Individuals Facing Social Stressors	Students	Employees and Others	Children and Adolescents
Residents Fellows Practicing physicians Registered dietitians Medical students Nursing students Physician assistant students Dental students	Patients experiencing overweight or obesity Patients with type 2 diabetes Breast cancer survivors Patients with kidney disease	WIC recipients Low-income individuals Patients in underserved areas Individuals experiencing food insecurity Individuals experiencing housing instability Veterans	College students Culinary students Health professional graduate students	Professional chefs Community-based leaders Workplace employees Families Older adults	Pediatric patients with inflammatory bowel disease Middle school students High school students

## Data Availability

No new data were created or analyzed in this study. Data sharing is not applicable to this article.

## Supplementary Materials

The following supporting information can be downloaded at: https://www.mdpi.com/article/10.3390/nu17233638/s1, Table S1: Descriptions of 36 Nutrients Articles. Table S2: Selected Additional Articles from Other Journals Evaluating the Impact of Teaching Kitchens and Related Interventions [48,49,50,51,52,53,54,55,56,57,58,59,60,61,62,63,64,65].

## Abbreviations

The following abbreviations are used in this manuscript:

FIM	Food is Medicine
US	United States
MAHA	Make America Healthy Again
GLP	Glucagon-like peptide
NIH	National Institutes of Health
ONR	Office of Nutrition Research
