# Supplementary material for: The Design and Impact of Teaching Kitchens and Hands-On Cooking Strategies on Diverse Populations: Increasing Evidence of Positive Effects and Proposed Future Directions
Source: Nutrients. 2025 Nov 21;17(23):3638. doi: 10.3390/nu17233638 (PMC12693395; doi:10.3390/nu17233638)
Supplement: Supplementary file 1 [file nutrients-17-03638-s001.zip › Nutrients Supplementary Table S2.pdf]

Supplementary Table S2. Selected Additional Articles from Other Journals Evaluating the Impact of Teaching Kitchens and Related Interventions.

Articles by Core Category	Brief Summary
**Populations**	
Health professionals and trainees	
Culinary medicine and community partnership: hands-on culinary skills training to empower medical students to provide patient-centered nutrition education[39]<br>Pang, B, Memel, Z, Diamant, C, et al. (2019)	This study demonstrates that a hands-on culinary medicine program improves cooking confidence and nutrition counseling skills for **medical students**.
Nutrition from the kitchen: culinary medicine impacts students' counseling confidence[40]<br>Magallanes, E., Sen, A., Siler, M. et al. (2021)	This study explores how a hands-on culinary medicine elective influences **first-year medical students'** comfort and skills in discussing nutrition with patients, as well as working with dietitians to support patients' lifestyle changes.
A Randomized Controlled Trial of a Culinary Medicine Intervention in a Virtual Teaching Kitchen for Primary Care Residents[41]<br>Wood NI, Fussell M, Benghiat E, et al. (2025)	This study illustrates that a virtual culinary medicine curriculum delivered to **primary care residents** leads to greater improvements in dietary counseling confidence compared to traditional didactic nutrition education.
Virtual teaching kitchen classes and cardiovascular disease prevention counselling among medical trainees[42]<br>Razavi AC, Latoff A, Dyer A, et al. (2023)	This study examines the impact of virtual culinary medicine education on **medical trainees'** dietary habits and counseling skills related to cardiovascular disease prevention and suggests that the virtual curriculum leads to improved adherence to Mediterranean diet guidelines and competency in lifestyle medicine counseling.
Adults with chronic conditions	
Mitigating Preventable Chronic Disease through the Cleveland Clinic Lifestyle 180 Program[43]<br>Ricanati EH, Golubi M, Yang D, et al. (2011)	This study illustrates that an intensive lifestyle intervention that includes hands-on culinary instruction for **adults with at least one chronic condition** leads to significant improvements in body weight, blood pressure, waist circumference, and emotional well-being.
Effects of Meal Preparation Training on Body Weight, Glycemia, and Blood Pressure in Type 2 Diabetes[44]<br>Dasgupta K, Hajna S, Joseph L, et al. (2012)	This study demonstrates that a 6-month culinary and lifestyle intervention among **adults with type 2 diabetes** leads to modest reductions in body weight and systolic blood pressure and improvements in meal planning confidence and dietary behaviors.
Impact of a Virtual Culinary Medicine Curriculum on Biometric Outcomes, Dietary Habits, and Related Psychosocial Factors among Patients with Diabetes Participating in a Food Prescription Program[45]<br>Sharma SV, McWhorter JW, Chow J, et al. (2021)	This study describes that a virtual culinary medicine program for **patients with diabetes** leads to significant reductions in HbA1c and improvements in cooking habits, dietary choices, and meal planning confidence.
A VA Health Care Innovation: Healthier Kidneys Through Your Kitchen-Earlier Nutrition Intervention for Chronic Kidney Disease[46]<br>Schlueter R, Calhoun B, Harned E, et al. (2021)	This study describes a nutrition education program which combines a single-session class with a cooking demonstration for **veterans with stage 3 chronic kidney disease,** and reveals significant knowledge gaps among participants, underscoring the need for continued education.

Cooking for Vitality: Pilot Study of an Innovative Culinary Nutrition Intervention for Cancer-Related Fatigue in Cancer Survivors[47]<br>Pritlove C, Capone G, Kita H, et al. (2020)	This study reveals that a culinary nutrition program for **cancer survivors** leads to improvements in cancer-related fatigue, energy levels, functional ability, and confidence in managing symptoms.
Influence of a Virtual Plant-Based Culinary Medicine Intervention on Mood, Stress, and Quality of Life Among Patients at Risk for Cardiovascular Disease[48]<br>Krenek AM, Aggarwal M, Chung ST, et al. (2025)	This study demonstrates that a 9-week virtual culinary medicine intervention for **adults at risk of cardiovascular disease** improves mood, reduces stress, and enhances quality of life.
Individuals facing social stressors	
Cooking Matters for Adults Improves Food Resource Management Skills and Self-confidence Among Low-Income Participants[49]<br>Pooler JA, Morgan RE, Wong K, et al. (2017)	This study highlights that the *Cooking Matters for Adults* curriculum delivered to **individuals from low-income backgrounds** leads to increased confidence in meal planning, budgeting, and preparation.
Employees	
Feasibility Pilot Study of a Teaching Kitchen and Self-Care Curriculum in a Workplace Setting[50]<br>Eisenberg DM, Righter AC, Matthews B, et al. (2017)	This study demonstrates that a 10-week teaching kitchen intervention delivered to **employees at a large U.S. workplace** leads to significant improvements in biometric measures, dietary habits, cooking confidence, and stress management.
A Culinary-Based Intensive Lifestyle Program for Patients with Obesity: The Teaching Kitchen Collaborative Curriculum (TKCC) Pilot Study[51]<br>McClure AC, Fenn M, Lebby S, et al. (2025)	This study demonstrates that a 6-week teaching kitchen intervention for **adult employees** at a health system leads to improvements in body weight, physical activity levels, cooking self-efficacy, and overall dietary behaviors.
Effectiveness of a Teaching Kitchen Intervention on Dietary Intake, Cooking Self-Efficacy, and Psychosocial Health[52]<br>Novotny D, Urich SM, Roberts HL (2022)	This study illustrates that a 5-week teaching kitchen program for **adult employees** leads to improvements in fruit, vegetable, and whole grain intakes, as well as increases in cooking confidence and overall well-being.
Dietary, Cooking, and Eating Pattern Outcomes from the Emory Healthy Kitchen Collaborative[53]<br>Moore MA, Wang D, Bergquist SH, et al. (2023)	This study demonstrates that a 12-month worksite wellness initiative delivered to **workplace employees** improves dietary patterns and mindful eating behaviors.
A Teaching Kitchen Program Improves Employee Micronutrient and Healthy Dietary Consumption[54]<br>Moore MA, Cousineau BA, Rastorguieva K, et al. (2023)	This study highlights the effectiveness of a 10-week teaching kitchen program in improving micronutrient adequacy and diet quality among **university and healthcare employees.**
Others	
Effect of culinary education curriculum on Mediterranean diet adherence and food cost savings in families: a randomised controlled trial[55]<br>Razavi AC, Sapin A, Monlezun DJ, et al. (2020)	This study illustrates that **families** that participate in a hands-on culinary education program are significantly more likely to embrace a Mediterranean-style diet and report cost savings compared to those receiving standard dietary counseling.
The effect of culinary medicine to enhance protein intake on muscle quality in older adults: a randomized controlled trial[56]<br>Galyean S, Alcorn M. Chavez J, et al. (2025)	This study suggests that a 16-week virtual culinary medicine intervention may support muscle health and nutrition in **older adult** populations.
